# MOP−18−Derived CuO Fiber for Hybrid Supercapacitor Electrodes

**DOI:** 10.3390/ma17061444

**Published:** 2024-03-21

**Authors:** Syed Fahad Bin Haque, Kenneth J. Balkus, John P. Ferraris

**Affiliations:** Department of Chemistry and Biochemistry, The University of Texas at Dallas, Richardson, TX 75080-3021, USA; syedfahadbin.haque@utdallas.edu (S.F.B.H.);

**Keywords:** hybrid supercapacitor, pseudocapacitance, metal−oxide fiber

## Abstract

This study explores a simple method of fabricating hybrid supercapacitor electrodes, which could potentially broaden the application of this technology. The method involves electrospinning a uniform solution of Matrimid/Metal−Organic Polyhedra 18 (MOP−18) followed by carbonization at a relatively low temperature of 700 °C in air, rather than in an inert atmosphere, to create free−standing, redox−active hybrid supercapacitor electrodes. Additionally, the synthesis procedure requires no stabilization or activation steps, which enhances the cost effectiveness of the synthesized electrode materials. The resulting C/CuO composite was used as the working electrode, with a polyacrylonitrile (PAN)/Poly(methyl methacrylate) (PMMA) carbon nanofiber (CNF) electrode as the counter and 6 M KOH as the electrolyte in a T−cell configuration. The cell performance and redox activity were evaluated using cyclic voltammetry (CV), galvanostatic charge–discharge (GCD), electrochemical impedance spectroscopy (EIS) and cycling stability tests. Additionally, the physical and chemical structures of the electrode materials were assessed using X−ray photoelectron spectroscopy (XPS), scanning electron microscopy (SEM), transmission electron spectroscopy (TEM), X−ray diffractometry (PXRD), surface area analysis and other characterization techniques. The electrode material demonstrated a specific capacitance of up to 206 F/g. Supercapacitors utilizing this material display an energy density of 10.3 Wh/kg (active material) at a current density of 1 A/g in electrochemical testing.

## 1. Introduction

The imminent surge in demand for effective and economical electrical energy storage systems, driven by the transition toward renewable energy sources and electric transportation, compels the exploration of innovative solutions [1,2]. Hybrid supercapacitors, which combine the features of electrochemical double−layer capacitors (EDLC) and pseudocapacitors (PC), are emerging as a promising alternative to meet the evolving energy storage requirements. These systems offer a blend of safety, affordability, durability and sustainability, often outperforming many other existing energy storage methods.

While Li−−ion batteries—the prevalent energy storage technology for consumer electronics and electric vehicles—have seen substantial enhancements in energy density and cost effectiveness, their limitations, such as restricted operating temperature range, low power density, limited cyclability and potential fire hazards, remain [3,4]. EDLCs, with their superior power density and extended cycle life, have been investigated as potential substitutes, despite their significantly lower energy density. PCs, which utilize conducting polymers and redox−active metal oxides, offer improved energy density compared to EDLCs, but their utility is constrained by poor cyclability or low electrical conductivity [5]. Hybrid supercapacitors integrate the strengths of both EDLCs and PCs and have the potential to deliver an optimal balance of high energy density, power density and cyclability [6,7,8,9,10,11].

The electrodes of hybrid supercapacitors typically comprise a composite or a physical mixture of carbon−based EDLC components and a pseudocapacitive component [12,13]. The EDLC component primarily contributes to conductivity and power density, while the pseudocapacitive materials enhance energy density. The primary challenge in designing hybrid supercapacitor electrodes lies in the integration of EDLCs and PCs in a manner, which allows each one to augment the other’s performance [6].

The interface between these two components significantly influences the performance and applicability of the electrode material. Pseudocapacitive materials, which are redox−active, require a high effective surface area, electrolyte accessibility and sufficient electrical conductivity to function optimally. A physical blend of carbon and redox−active materials often falls short of providing these necessities to the redox−active species. Therefore, specialized structures, such as core–shell, 1D and 2D hybrids, intimate contact composites and advanced mixing techniques, such as in situ dispersion, are essential for both components to operate optimally [14,15,16,17,18,19,20,21,22]. Electrospinning, an in situ dispersion technique, was utilized in this study to prepare uniform composite fiber mats from PC and EDLC precursors, followed by carbonization to prepare the electrodes.

The primary focus of this study is to understand the viability of using Metal−Organic Framework−18 (MOP−18) as a precursor to pseudocapacitive CuO nanoparticles to be used in hybrid supercapacitor electrodes. MOP−18 has an inner core comprising 24 copper atoms arranged in 12 paddle−wheel units. The organic outer layer has 24 dodecyl groups attached to it [23,24]. This long alkyl chain outer structure gives MOP−18 exceptional solubility in several organic solvents, ensuring a uniform dispersion in the fibers produced by the electrospinning process. The small copper core of MOP−18 could serve as a potential source of redox−active particles with very small cluster sizes and large surface area. During the process of carbonization, polymeric EDLC precursors are transformed into carbon materials, providing conductivity and mechanical strength to the composite electrode. The commercially available polyamide Matrimid was used as the MOP−18 carrier in the electrospinning process and as a potential EDLC precursor. Supercapacitor test cells utilizing working electrodes punched out from the free−standing carbonized fiber mats were characterized using numerous electrochemical techniques, including cyclic voltammetry (CV), galvanostatic charge–discharge (GCD) and electrochemical impedance spectroscopy (EIS).

## 2. Materials and Methods

### 2.1. Materials

Matrimid 5218, procured from Ciba Specialty Chemicals, was used as received. Potassium hydroxide was purchased from Fisher Scientific, Waltham, MA, USA. Sodium bicarbonate, 5−hydroxyisophthalic acid, 1−iodododecane, ethyl acetate, acetonitrile, methanol, copper acetate monohydrate, tetrachloroethane and chloroform were procured from Sigma−Aldrich, St. Louis, MO, USA, with purities ≥97% and used without additional purification. Dimethylformamide (DMF) and dimethylacetamide (DMAc), both 99.8% pure, were sourced from Sigma−Aldrich and Millipore Corporation, Burlington, MA, USA, respectively.

### 2.2. MOP−18 Synthesis

The synthesis of MOP−18 was carried out following a previously established protocol [23]. Briefly, the linker for MOP−18 synthesis, 5−dodecyloxyisophthalic acid, was prepared via esterification of 5−hydroxyisophthalic with ethanol, followed by alkylation of the diester’s hydroxyl group and subsequent hydrolysis. A solution was prepared by dissolving 2.7 g of the synthesized linker in 100 mL of DMF at 80 °C with continuous stirring until fully dissolved. Concurrently, a solution of 1.5 g of copper acetate in 50 mL of DMF was prepared separately at room temperature. The two solutions were combined, and 100 mL of methanol was introduced to initiate precipitation. The resulting solid precipitate was allowed to crystallize for 24 h at room temperature, followed by repeated washing with methanol. The resultant blue solid was dried at 60 °C for 24 h under vacuum. The final product—MOP−18 crystals—was subsequently characterized using powder XRD.

### 2.3. Electrospinning

Matrimid (0.6 g) was dissolved in 3.63 mL of DMAc at ambient temperature to yield a 15% *w/w* solution. Concurrently, a 15 wt.% solution of MOP−18 was prepared by dissolving 1.2 g of MOP−18 in 6.8 mL of DMAc at room temperature. Subsequently, the Matrimid solution and MOP−18 solution were combined to produce 1:2 dry *w/w* ratios of Matrimid/MOP−18. The mixtures were stirred for 24 h at room temperature to ensure homogeneity.

The resultant solution was then subjected to electrospinning using a custom−built electrospinning setup. The homogeneous polymer solution was dispensed at a rate of 0.5 mL/h. The 20 Ga blunt tip needle, connected to the 10 mL syringe, was maintained at a positive potential of 20 kV with a reciprocating translational motion during spinning. A collecting drum, covered with aluminum foil, was maintained at a negative potential of 5 kV and rotated at 300 rpm. The distance between the needle tip and the collector was kept constant at 15 cm. The as−spun fibers were collected and dried under a vacuum at 150 °C for 24 h. To carbonize the e−spun fibers, the mat was heated at 700 °C under air using a heating rate of 2 °C/min and maintained at this temperature for 60 min. The resulting carbonized 1:2 Matrimid/MOP−18 fibers will be referred to as MM12 in the following sections.

### 2.4. Instrumentation

Several characterization techniques were employed to elucidate the physical structure and electrochemical performance of the synthesized Matrimid/MOP−18 hybrid electrodes. Powder X−ray diffraction patterns were obtained using a Rigaku (Woodlands, TX, USA) Ultima IV diffractometer, employing Cu Kα radiation. Raman spectra were acquired using a Thermo Scientific (Waltham, MA, USA) DXR Raman microscope, with a laser excitation wavelength of 532 nm. Surface elemental composition was determined through X−ray photoelectron spectroscopy (XPS) measurements, conducted using a PHI (Chanhassen, MN, USA) Versa Probe II, with monochromatic Al Kα radiation (hv = 1486.6 eV) employed to generate the photoelectrons. The spectra were collected in a hemispherical analyzer at a step size of 0.2 eV and a pass energy of 11 eV for the survey and 23.5 eV for the analysis. SEM micrographs of carbonized samples were collected using a Zeiss (Oberkochen, Germany) SUPRA 40 SEM instrument. The morphology of these fibers was further studied using transmission electron microscopy (HRTEM) on a JEOL (Peabody, MA, USA) JEM−1400 TEM at 120 kV. Nitrogen and carbon dioxide adsorption−desorption isotherms at 77 K and 273 K, respectively, were obtained using Micromeritics (Norcross, GA, USA) ASAP 2020. The specific surface area post−carbonization was determined using the Brunauer−Emmett−Teller (BET) method. Pore size distribution was ascertained from the gas adsorption–desorption isotherm using the two−dimensional non−linear density functional theory (2D NLDFT) method. The TA Instruments (New Castle, DE, USA) SDT Q600 was used for thermogravimetric analysis (TGA)

## 3. Results and Discussion

### 3.1. Material Characterization

**Figure 1:**
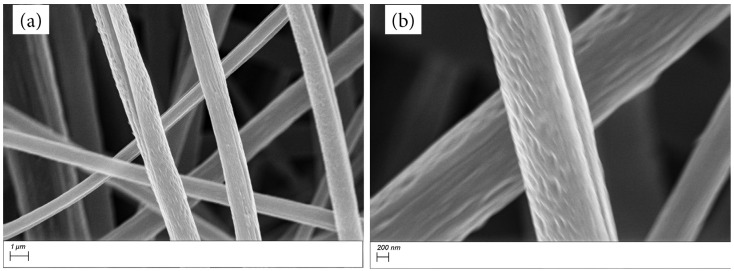
SEM images of as−spun 1:2 Matrimid/MOP−18 fibers with a (**a**) 1 µm, (**b**) 200 nm scale bar.

**Figure 2:**
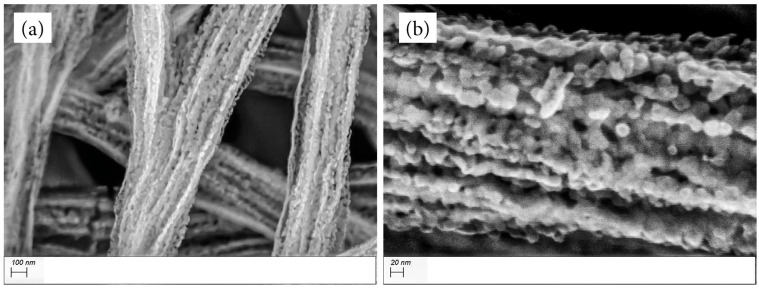
SEM images of MM12 fibers with a (**a**) 100 nm, (**b**) 20 nm scale bar.

SEM images in Figure 1 show the morphology of 1:2 Matrimid/MOP−18 fibers before carbonization. The fibers have average diameters of 1.27 µm with small indentations on their surfaces. This morphology changes dramatically upon carbonization in air, as shown in the SEM images of MM12 in Figure 2. The fibers now predominantly consist of interconnected particles. The carbonization process conducted in air causes oxidative loss of carbon from Matrimid and MOP−18, resulting in 2% residual carbon in MM12 and concomitant formation of CuO (vide infra). The average diameter of the fibers after carbonization is 450 nm. The optimal conditions for this process (700 °C, 1 h) were determined through control experiments to ensure sufficient residual carbon in the structure to bind the particles into a fibrous form. This is crucial for maintaining the surface area, the self−supporting nature of the electrode and the accessibility of the electrolyte to the particles. Carbonization exceeding 700 °C in air compromises the mechanical strength of the electrode material, rendering it unsuitable for use as self−supporting electrodes. Conversely, carbonization at temperatures below 700 °C causes incomplete conversion of Cu metal particles to CuO and yields a mixture of different oxidation states of Cu.

**Figure 3:**
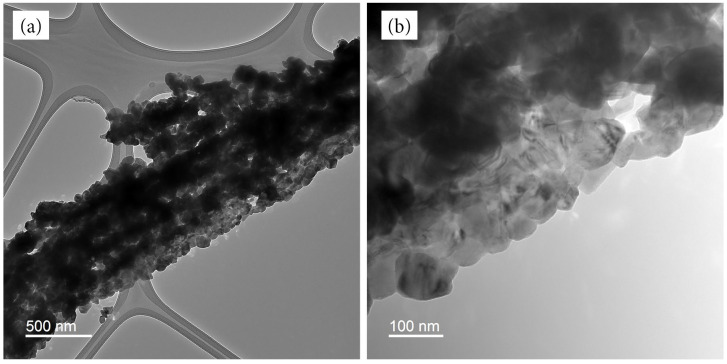
TEM images of MM12 fibers with a (**a**) 500 nm, (**b**) 100 nm scale bar.

Figure 3 shows TEM images of MM12 after micronization. Most of the fibers are composed of nanoparticles held together by a minimum amount of carbon. This is in contrast to most reported studies of pseudocapacitive redox−active particle−impregnated carbon fiber structures, wherein the fibers consist predominantly of carbon [25,26]. The carbon in MM12 serves as a binder to maintain the cohesion of the particles into a fibrous morphology. This observation agrees with the results obtained from SEM and XPS shown below. These data suggest that the composite’s capacitance will be primarily derived from pseudocapacitance, as opposed to electrochemical double−layer capacitance.

**Figure 4:**
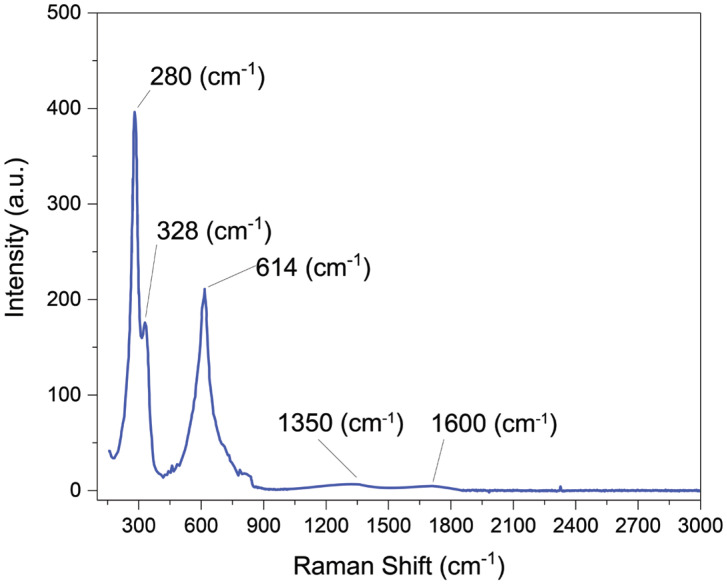
Raman spectra of MM12.

Figure 4 presents a Raman spectrum of MM12 fibers. Three characteristic peaks of CuO nanoparticles are visible in the Raman spectra at 280 cm^−1^, 328 cm^−1^ and 614 cm^−1^ [27,28]. The responses of CuO at these wavelengths agree with the reported literature. Two very small peaks of carbon are also observable close to 1350 cm^−1^ and 1600 cm^−1^ ascribed to the D and G peaks of graphite [29].

**Figure 5:**
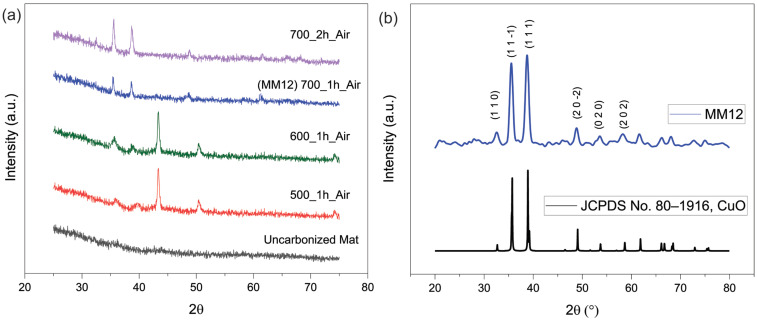
(**a**) X−ray diffraction comparison between the uncarbonized mat and different carbonization temperatures and times; (**b**) High−resolution X−ray diffraction of MM12.

An X−ray diffraction comparison between mats carbonized at different temperatures and for different times along with uncarbonized as−spun samples is presented in Figure 5a. It is evident that MOP−18 fully oxidizes into CuO particles at 700 °C, and below that temperature, the materials consist of a mixture of CuO, Cu and Cu_2_O. Notably, a 2 h soak time at 700 °C converted the fiber mat into CuO powder, which could no longer be used as a free−standing electrode material. Hence, to achieve complete conversion to CuO and maintain the free−standing nature of the electrode materials, a 1 h soak time at 700 °C carbonization temperature was utilized, which is indicated in Figure 5a with the blue line. Figure 5b presents a higher resolution X−ray diffraction spectrum of Matrimid/MOP−18 fibers after carbonization. To better visualize the peaks, a slower scan rate was utilized to characterize the MM12 sample. The X−ray diffraction pattern encompasses all the significant peaks of CuO. Noteworthy peaks are evident at 2θ values of 32.7°, 35.7°, 38.9° and 48.1°, corresponding to the crystal phases of (1 1 0), (1 1 −1), (1 1 1) and (2 0 −2) of CuO, respectively, and agree with the XRD pattern at JCPPDS card no. 80−1916 of CuO [30,31]. The absence of peaks at 2θ values of 43.5°, 50.4° and 74.2° (JCPDS No. 003–1018) signifies the complete conversion of Cu particles from MOP−18 to CuO during the carbonization process in air [32]. XRD peaks associated with Cu_2_O at 2θ of 36.3°, 42.4° and 61.6° (JCPDS No. 05−0667) are also absent from the spectra [33].

**Figure 6:**
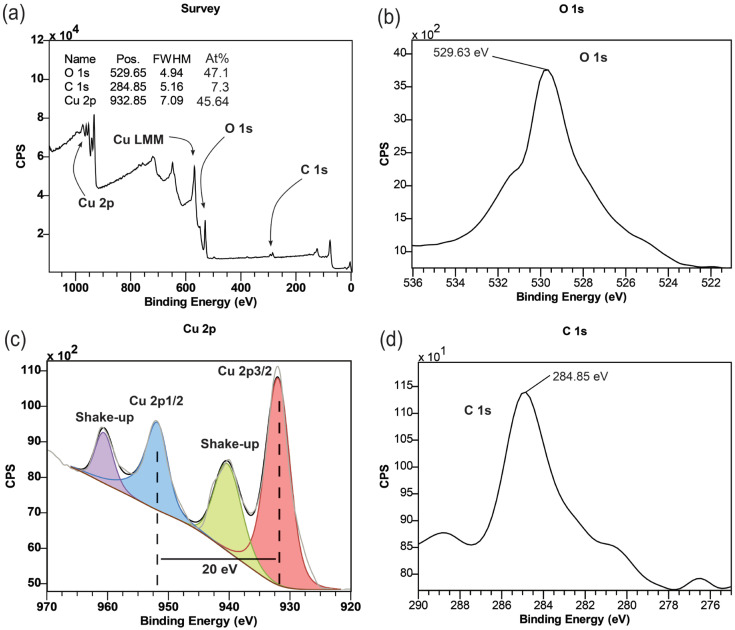
XPS spectra of MM12 fibers: (**a**) Survey, (**b**) O 1s, (**c**) Cu 2p and (**d**) C 1s.

The X−ray photoelectron spectra of MM12 fibers are presented in Figure 6. A gas cluster ion beam (GCIB) gun mounted on the vacuum chamber of the XPS instrument was used for 60 s to clean up and prepare sample surfaces before collecting XPS spectra. A charge neutralization electron beam gun was used to prevent surface charge accumulation. The survey spectra, depicted in Figure 6a, reveal the presence of copper, oxygen and carbon atoms. The relative intensity of these elements allowed for the calculation of their respective masses, which were found to be 77.84% for copper, 19.83% for oxygen and 2.33% for carbon. The atomic ratio of oxygen to copper was determined to be 1.03:1, indicating that the copper from MOP−18 was oxidized to CuO. Figure 6b,d show narrow scans of O 1s and C 1s XPS spectra, showing peaks associated with oxygen at 529.65 eV and carbon at 932.85 eV, respectively. The low signal−to−noise ratio of carbon peaks caused by their relative scarcity (<2%) prevented further peak fitting to reveal the oxidation state of carbon atoms present in the composite [34,35]. Figure 6c displays a high−resolution selected area scan for copper. The peaks for Cu 2p3/2 and Cu 2p 1/2 occurred at 933 and 953 eV, respectively, with a separation of 20 eV [36]. Shake−up satellite peaks for both Cu 2p 3/2 and 2p 1/2 were observed at approximately 9 eV higher binding energy [37]. The shape and peak positions of the copper spectra and their shake−ups are consistent with the spectra of CuO [38,39,40].

**Figure 7:**
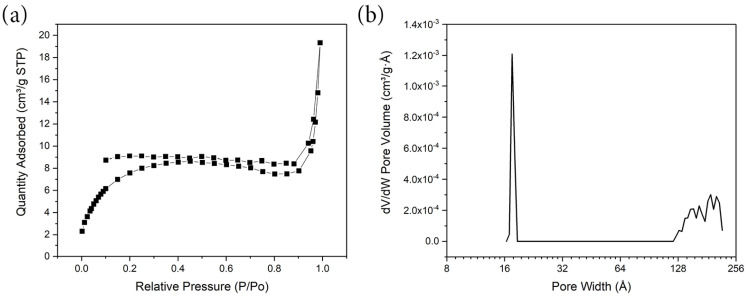
(**a**) N_2_ adsorption–desorption isotherm and (**b**) pore size distribution of MM12.

Figure 7 illustrates the nitrogen adsorption–desorption isotherm for micronized MM12. Before the analysis at 77 K, the sample was degassed for 24 h at a temperature of 250 °C. The MM12 fibers exhibited a type IV gas adsorption–desorption isotherm, characterized by a relatively minor hysteresis [41]. Both the pore size distribution and the gas adsorption–desorption isotherm suggest a low porosity. Given that more than 98% of the material comprises non−porous CuO nanoparticles, the specific surface area was restricted to 40 m^2^/g. The primary pore systems are observable within a range of 1.5–1.7 nm. A minor quantity of mesopores was also discernible within the 12–25 nm regions. The electrode material’s deficiency in microporosity resulted in its limited performance in terms of electric double−layer capacitance (EDLC) in organic or ionic liquid electrolytes. Therefore, the primary focus of this work explored MM12′s behavior in an aqueous electrolyte.

**Figure 8:**
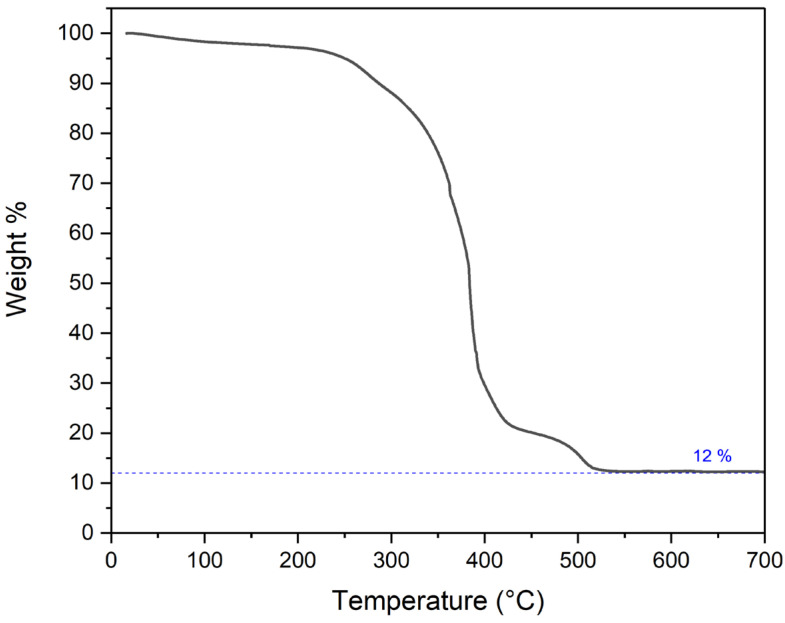
Thermogravimetric analysis of as−spun 1:2 Matrimid/MOP−18 fibers in air.

**Figure 9:**
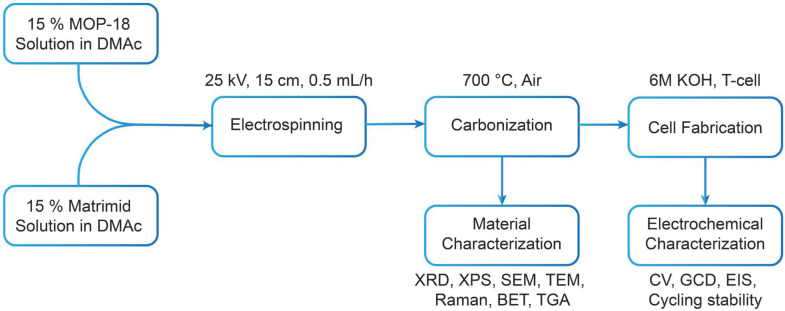
Flow chart of the synthesis procedure of MM12 and successive characterization and electrochemical testing.

Figure 8 presents a thermogravimetric analysis of the as−spun 1:2 Matrimid/MOP−18 fiber in an air environment. The heating protocol replicated the carbonization process employed in this research. The temperature was ramped at a rate of 2 °C per min. A significant mass loss, accounting for 88% of the total, was observed within the temperature range of 300–500 °C. This mass reduction can be attributed to the decomposition of Matrimid and the thermal degradation of MOP−18. It has been reported that MOP−18 undergoes thermal decomposition between 300 °C and 450 °C [42]. During the thermal decomposition phase of MOP−18, Cu crystals, when exposed to high temperatures and air, oxidize to form CuO. Consequently, at a carbonization temperature of 700 °C, only 12% of the initial mass remains, predominantly composed of CuO particles. These particles are oriented in a fibrous formation, held together by a minimal amount of residual carbon. Figure 9 shows a flow chart diagram demonstrating the preparation and characterization process utilized in this study.

### 3.2. Electrochemical Characterization

**Figure 10:**
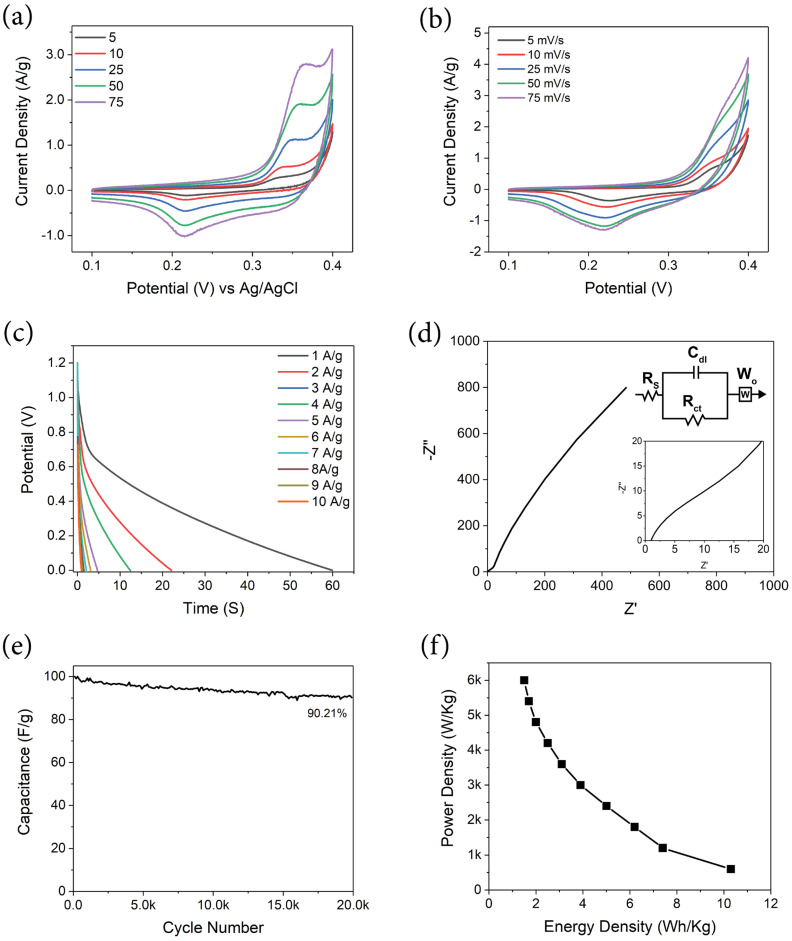
(**a**) Three−electrode cyclic voltammetry of MM12 vs. Ag/AgCl reference electrode in saturated KCl. (**b**) Two−electrode cyclic voltammetry of MM12. (**c**) Galvanostatic charge–discharge of MM12. (**d**) Nyquist plot of electrochemical impedance spectroscopy of MM12. (**e**) Cyclability experiment of MM12 fibers at 1 A/g. (**f**) Ragone plot of MM12 fibers from the galvanostatic charge–discharge experiment.

The electrochemical analysis of all electrode materials was performed using T cells, with the MM12 fiber mats functioning as the working electrode. As the MM12 mat is free−standing, 1 cm^2^ discs of the working electrode were punched out directly from the mat and were used without any additional binder or conducting additives. An asymmetric cell was assembled utilizing previously reported carbonized and CO_2_−activated 4:1 PAN/PMMA fiber as the counter electrode [43]. A 1 cm^2^ disc of the counter electrode was punched out similarly from the free−standing mat and was used as obtained. The electrodes were separated by a PTFE membrane. An Ag/AgCl electrode in a saturated KCl solution was used as the reference electrode. A 6 M KOH solution was used as the electrolyte. Mass ratios of working and counter electrodes were carefully adjusted to maintain the charge balance in each cell during fabrication. The electrochemical data presented in Figure 10 were obtained from a T cell fabricated with a working electrode of 1.25 mg and a counter electrode of 1.87 mg to balance the charge. Each cell prepared was cycled 50 times at a 50 mV/s scan rate in a cyclic voltammetry protocol for activation. All data presented in Figure 10 were taken after this activation step.

Figure 10a illustrates cyclic voltammetry (CV) at multiple scan rates over a potential range of 0.1 V–0.4 V in a three−electrode setup. The redox couple associated with CuO is visible, with an oxidation peak at 0.35 V and a reduction peak at 0.19 V. These features retain their relative position with increasing scan rate from 5 mV/s to 75 mV/s, indicating a fast reversible redox reaction. The reactions mentioned below illustrate the charge storage mechanism of CuO in KOH electrolytes [44,45,46,47]. The anodic peaks are not unique because the oxidation processes from Cu_2_O or CuOH to CuO and Cu(OH)_2_, respectively, overlap [48].

$$2\mathrm{CuO}+H_{2}O+2e^{-}↔{\mathrm{Cu}}_{2}O+2{\mathrm{OH}}^{-}$$

$${\mathrm{Cu}}_{2}O+{\;H}_{2}O+2{\mathrm{OH}}^{-}↔2\mathrm{Cu}{\left(\mathrm{OH}\right)}_{2}+2e^{-}$$

$$2\mathrm{CuOH}+{\;\mathrm{OH}}^{-}↔2\mathrm{Cu}{\left(\mathrm{OH}\right)}_{2}+2e^{-}$$

$$\mathrm{CuOH}+{\mathrm{OH}}^{-}↔\mathrm{CuO}+H_{2}O+e^{-}$$

Figure 10b displays a two−electrode cyclic voltammogram of MM12 working negative electrode vs. PAN: PMMA counter positive electrode at scan rates of 5, 10, 25, 50 and 75 mV/s [49,50,51,52,53]. The voltammograms exhibit similar broad peaks resulting from redox activity seen in three−electrode experiments. The peaks resulting from redox activity visible in the CVs complicate the calculation of capacitance, energy density and power density from the data obtained through the method. Hence, galvanostatic charge–discharge experiments were used to compute these performance metrics presented in Table 1.

Figure 10c presents the galvanostatic charge–discharge (GCD) of MM12 working negative electrode vs. PAN: PMMA counter positive electrode from 1–10 A/g current density. The GCD experiment was conducted between 1.2 V and 0 V. The electrochemical performance values of the electrode materials were derived from GCD using Equations (1)–(4) and are presented in Table 1 [54,55]. The MM12 electrode material demonstrated a specific capacitance of up to 206 F/g. Supercapacitors utilizing this material display an energy density of 10.3 Wh/kg (active materials) at a current density of 1 A/g in electrochemical testing. A comparison of the performance matrices between this work and previously reported studies is presented in Table 2.

The relative scarcity of carbon and low surface area of 40 m^2^/g cause the electrochemical kinetics to be diffusion−controlled rather than capacitive. The equations utilized to calculate the specific capacitance, energy density and power density are shown below in Equations (1)–(4).

(1) $$C_{cell}=\frac{I\;∆t}{m\;∆V}$$

(2) $$C_{electrode}=4C_{cell}$$

(3) $$E=\frac{1}{2}\times \frac{C_{cell}\;∆V^{2}}{3600}\times 1000$$

(4) $$P=\frac{E}{∆t}\times 3600$$

In Equation (1), “*C_cell_*” is the cell capacitance, and in Equation (2), “*C_electrode_*” is the specific gravimetric capacitance of the working electrode only; “∆t” is the time of discharge in seconds; “*I*/*m*” is the current in amperes divided by the total electrode mass in grams, which implies the current density; and “∆V” is the potential window in volts. In Equation (3), “*E*” is the energy density in Wh/kg, and in Equation (4), “*P*” is the power density in W/kg [55].

Electrochemical impedance spectroscopy (EIS), as illustrated in Figure 10d as a Nyquist plot, encompasses a frequency spectrum ranging from 100 kHz to 0.01 Hz, with a specific focus on the high−frequency domain and an equivalent circuit model, as highlighted in the inset. The intersection point of the EIS spectra with the X−axis signifies the electrolyte resistance, denoted as *R_s_*, while the semicircle in the immediately lower frequency represents the charge transfer resistance of pseudocapacitance, denoted as *R_ct_* in the equivalent circuit model [62]. In the instance of MM12, the *R_s_* was 1.044 Ω, and the cell did not display a discernible high−frequency semicircle, as the *R_ct_* was 3.5 Ω. The diminished electrolyte resistance and absence of a conspicuous semicircle can be ascribed to the high conductivity of 6 M KOH and low interfacial impedance between cell components [63]. Additionally, the rapid redox reaction of CuO particles, observable in CVs, is presumably not a significant contributor to the impedance. As demonstrated in previous research, CuO−carbon composites with adequate conductivity exhibit a Nyquist plot without a semicircular feature at a high−frequency region corresponding to the charge transfer resistance in electrochemical impedance spectroscopy [64,65]. The absence of capacitive components in a significant quantity and the low surface area of the electrodes impede the formation of a vertical segment in the low−frequency region indicative of capacitive behavior [66,67]. The straight line in the low−frequency region of the Nyquist plot is caused by Warburg diffusion impedance and hence fitted with a Warburg component denoted as W_0_ in the equivalent circuit model. The Nyquist plot in the low−frequency region reveals diffusion−controlled kinetics [45,68]. Consequently, the aggregate internal resistance (Rs + Rct) is relatively low, as evidenced by the EIS spectra compared to other reported studies of CuO−based hybrid supercapacitors [66,69,70].

Figure 10e illustrates the capacitance retention vs. cycle numbers for the MM12 composite cell. The experiment was conducted at a current density of 1 A/g in a galvanostatic charge–discharge protocol, with the cell undergoing repeated charging and discharging between 1.2 V and 0.6 V. The cycling stability test reveals that the composite electrode retains over 90% capacitance after 20,000 cycles, indicating superior cyclability of the electrode material compared to other studies of similar electrode systems [71,72,73].

Figure 10f presents a graph of power density vs. energy density, derived from a two−electrode galvanostatic charge–discharge experiment, with MM12 being the negative working electrode and PAN: PMMA being the counter positive electrode.

## 4. Conclusions

In summary, a relatively simple synthesis procedure of a CuO particle/carbon composite fiber via electrospinning 1:2 Matrimid/MOP−18 solution in DMAc with subsequent carbonization of the as−spun fibers in the air at 700 °C was devised. Using MOP−18 instead of other copper precursors helped ensure a uniform dispersion of nanoparticles of small dimensions. An energy density of 10.3 Wh/kg at a current density of 1 A/g was achieved in a 6 M KOH electrolyte, which is higher than the majority of the reported studies of similar electrode systems [70,71]. The method used in the study successfully illustrates the potential of materials such as metal−organic polyhedra as precursors for redox−active particles to be used in electrical energy storage.

## Figures and Tables

**Table 1:** Electrochemical performance of MM12 in the galvanostatic charge–discharge experiment.

Current Density (A/g)	Specific Capacitance (F/g)	Energy Density (Wh/kg)	Power Density (W/kg)
1	206.2	10.3	600
2	148.2	7.4	1200
3	124.5	6.2	1800
4	101.0	5.0	2400
5	77.5	3.9	3000
6	61.9	3.1	3600
7	49.2	2.5	4200
8	39.7	2.0	4800
9	34.2	1.7	5400
10	29.5	1.5	6000

**Table 2:** Comparison of electrochemical performance between this work and the literature.

Material	Electrolyte	Energy Density (W h kg ^−1^)	Power Density (kW kg ^−1^)	Ref.
CuO−activated charcoal	1.0 M NaOH	2.28	0.62	[56]
CuO nanosheet/AC	6.0 M KOH	5.59	0.84	[57]
CuO/Co_3_O_4_ MWCNT	5.0 M KOH	7.54	0.25	[58]
Activated charcoal–CuO (1:1)	6.0 M KOH	4.15	0.65	[59]
CuO−NiO nanocomposites	1.0 M H_2_SO4	3.14	0.51	[60]
CuO nanosheet/rGO	6 M KOH	3.5	0.2	[61]
This work	6 M KOH	10.3	0.6	

## Data Availability

Data are contained within the article.

## References

[1] Simon P., Gogotsi Y. (2020). Perspectives for Electrochemical Capacitors and Related Devices. Nat. Mater..

[2] Muzaffar A., Ahamed M.B., Deshmukh K., Thirumalai J. (2019). A Review on Recent Advances in Hybrid Supercapacitors: Design, Fabrication and Applications. Renew. Sustain. Energy Rev..

[3] Campbell P.G., Merrill M.D., Wood B.C., Montalvo E., Worsley M.A., Baumann T.F., Biener J. (2014). Battery/Supercapacitor Hybrid via Non-Covalent Functionalization of Graphene Macro-Assemblies. J. Mater. Chem. A.

[4] Yu H., Fan L., Wu J., Lin Y., Huang M., Lin J., Lan Z. (2012). Redox-Active Alkaline Electrolyte for Carbon-Based Supercapacitor with Pseudocapacitive Performance and Excellent Cyclability. Rsc Adv..

[5] Liew C.-W., Ramesh S. (2014). Comparing Triflate and Hexafluorophosphate Anions of Ionic Liquids in Polymer Electrolytes for Supercapacitor Applications. Materials.

[6] Simon P., Gogotsi Y., Dunn B. (2014). Where Do Batteries End and Supercapacitors Begin?. Science.

[7] Dubal D.P., Ayyad O., Ruiz V., Gómez-Romero P. (2015). Hybrid Energy Storage: The Merging of Battery and Supercapacitor Chemistries. Chem. Soc. Rev..

[8] Tian Y., Abbas M., Haque S.F.B., Zhu X., Ferraris J.P., Balkus K.J. (2024). RuxV2− XO5 Nanowire/Templated Carbon Composite Electrodes for Supercapacitors. Next Mater..

[9] Yu F., Zhou L., You T., Zhu L., Liu X., Wen Z. (2017). Preparation of Zn0. 65Ni0. 35O Composite from Metal-Organic Framework as Electrode Material for Supercapacitor. Mater. Lett..

[10] Yu F., Wang Y., Liu Y., Hui H.-Y., Wang F.-X., Li J.-F., Wang Q. (2022). An Aqueous Rechargeable Zinc-Ion Battery on Basis of an Organic Pigment. Rare Met..

[11] Li L., Wang Y., Meng J., Shen N., Liu H., Guo C., Bao W., Li J., Yao D., Yu F. (2023). Boosting the Capacitance of MOF-Derived Carbon-Based Supercapacitors by Redox-Active Bromide Ions. Chem. Eng. J. Adv..

[12] Zakharov A., Tukesheva A., Haque S.F.B., Ferraris J., Zakhidov A., Tazhibayeva T., Bazarbayeva T., Pavlenko V. (2023). Review of the Current State of Technology for Capacitive Deionization of Aqueous Salt Solutions. Bull. Karaganda Univ. Phys. Ser..

[13] Ayaganov Z., Pavlenko V., Haque S.F.B., Tanybayeva A., Ferraris J., Zakhidov A., Mansurov Z., Bakenov Z., Ng A. (2024). A Comprehensive Study on Effect of Carbon Nanomaterials as Conductive Additives in EDLCs. J. Energy Storage.

[14] Shi P., Li L., Hua L., Qian Q., Wang P., Zhou J., Sun G., Huang W. (2017). Design of Amorphous Manganese Oxide@ Multiwalled Carbon Nanotube Fiber for Robust Solid-State Supercapacitor. ACS Nano.

[15] Patil S.J., Chodankar N.R., Pujari R.B., Han Y.-K., Lee D.W. (2020). Core-Shell Hetero-Nanostructured 1D Transition Metal Polyphosphates Decorated 2D Bimetallic Layered Double Hydroxide for Sustainable Hybrid Supercapacitor. J. Power Sources.

[16] Bhoyate S., Kahol P.K., Gupta R.K. (2020). Broadening the Horizon for Supercapacitor Research via 2D Material Systems. Nanoscience.

[17] Hu B., Li H., Liu A., Yue C., Guo Z., Mu J., Zhang X., Che H. (2020). Construction of 2D–2D Plate-on-Sheet Cobalt Sulfide–Reduced Graphene Oxide Nanocomposites for Enhanced Energy Storage Properties in Supercapacitors. ACS Appl. Energy Mater..

[18] Yang C.-H., Hsiao Y.-C., Lin L.-Y. (2021). Novel In Situ Synthesis of Freestanding Carbonized ZIF67/Polymer Nanofiber Electrodes for Supercapacitors via Electrospinning and Pyrolysis Techniques. ACS Appl. Mater. Interfaces.

[19] Zhou J., Liu B.-B., Zheng H., Ma W.-Q., Li Q., Xu C.-X. (2024). One-Step Construction of Strongly Coupled Co_3_V_2_O_8_/Co_3_O_4_/MXene Heterostructure via in-Situ Co-F Bonds for High Performance All-Solid-State Asymmetric Supercapacitors. Rare Met..

[20] Peng M., Wang L., Li L., Peng Z., Tang X., Hu T., Yuan K., Chen Y. (2021). Molecular Crowding Agents Engineered to Make Bioinspired Electrolytes for High-Voltage Aqueous Supercapacitors. EScience.

[21] Wang T., Xiong C., Zhang Y., Wang B., Xiong Q., Zhao M., Ni Y. (2023). Multi-Layer Hierarchical Cellulose Nanofibers/Carbon Nanotubes/Vinasse Activated Carbon Composite Materials for Supercapacitors and Electromagnetic Interference Shielding. Nano Res..

[22] Xiong C., Wang T., Zhou L., Zhang Y., Dai L., Zhou Q., Ni Y. (2023). Fabrication of Dual-Function Conductive Cellulose-Based Composites with Layered Conductive Network Structures for Supercapacitors and Electromagnetic Shielding. Chem. Eng. J..

[23] Furukawa H., Kim J., Plass K.E., Yaghi O.M. (2006). Crystal Structure, Dissolution, and Deposition of a 5 Nm Functionalized Metal-Organic Great Rhombicuboctahedron. J. Am. Chem. Soc..

[24] Tajik M., Bin Haque S.F., Perez E.V., Vizuet J.P., Firouzi H.R., Balkus K.J., Musselman I.H., Ferraris J.P. (2023). Pillared Carbon Membranes Derived from Cardo Polymers. Nanomaterials.

[25] Samuel E., Aldalbahi A., El-Newehy M., El-Hamshary H., Yoon S.S. (2022). Flexible and Freestanding Manganese/Iron Oxide Carbon Nanofibers for Supercapacitor Electrodes. Ceram. Int..

[26] Anand S., Choudhury A. (2023). MnMoS4 Anchored at Carbon Nanofiber as a Flexible Electrode for Solid-State Asymmetric Supercapacitor Device. Mater. Chem. Phys..

[27] Rashad M., Rüsing M., Berth G., Lischka K., Pawlis A. (2013). CuO and Co_3_O_4_ Nanoparticles: Synthesis, Characterizations, and Raman Spectroscopy. J. Nanomater..

[28] Al Baroot A., Alheshibri M., Drmosh Q.A., Akhtar S., Kotb E., Elsayed K.A. (2022). A Novel Approach for Fabrication ZnO/CuO Nanocomposite via Laser Ablation in Liquid and Its Antibacterial Activity. Arab. J. Chem..

[29] Ferrari A.C. (2007). Raman Spectroscopy of Graphene and Graphite: Disorder, Electron-Phonon Coupling, Doping and Nonadiabatic Effects. Solid State Commun..

[30] Davaria S., Ramezanpoura S. (2018). Rapid Synthesis of a Nano-Sized Copper (II) Oxide by Calcination of the Cu (II) Schiff Base Complex. Chem. Methodol..

[31] Yang C., Xu H., Shi J., Liu Z., Zhao L. (2021). Preparation and Photocatalysis of CuO/Bentonite Based on Adsorption and Photocatalytic Activity. Materials.

[32] Liu A., Shi Z., Reddy R.G. (2020). Mechanism Study of Cu-Zn Alloys Electrodeposition in Deep Eutectic Solvents. Ionics.

[33] Yang Z., Zhang D., Zhang W., Chen M. (2009). Controlled Synthesis of Cuprous Oxide Nanospheres and Copper Sulfide Hollow Nanospheres. J. Phys. Chem. Solids.

[34] Stickle W., Stickle T. (2014). Propylene Carbonate-XPS Reference Spectra. Surf. Sci. Spectra.

[35] Leiro J.A., Heinonen M.H., Laiho T., Batirev I.G. (2003). Core-Level XPS Spectra of Fullerene, Highly Oriented Pyrolitic Graphite, and Glassy Carbon. J. Electron Spectros. Relat. Phenomena.

[36] Yuan J., Zhang J.-J., Yang M.-P., Meng W.-J., Wang H., Lu J.-X. (2018). CuO Nanoparticles Supported on TiO_2_ with High Efficiency for CO2 Electrochemical Reduction to Ethanol. Catalysts.

[37] Ibupoto Z.H., Tahira A., Raza H., Ali G., Khand A.A., Jilani N.S., Mallah A.B., Yu C., Willander M. (2018). Synthesis of Heart/Dumbbell-like CuO Functional Nanostructures for the Development of Uric Acid Biosensor. Materials.

[38] Sakai Y., Ninomiya S., Hiraoka K. (2012). XPS Depth Analysis of CuO by Electrospray Droplet Impact. Surf. interface Anal..

[39] Chang S.-S., Lee H.-J., Park H.J. (2005). Photoluminescence Properties of Spark-Processed CuO. Ceram. Int..

[40] Malik M.A., Surepally R., Akula N., Cheedarala R.K., Alshehri A.A., Alzahrani K.A. (2022). Oxidation of Alcohols into Carbonyl Compounds Using a CuO@ GO Nano Catalyst in Oxygen Atmospheres. Catalysts.

[41] Frikha K., Limousy L., Bouaziz J., Chaari K., Bennici S. (2020). Synthesis, Characterization and Catalytic Activity of Ternary Oxide Catalysts Using the Microwave-Assisted Solution Combustion Method. Materials.

[42] Perez E.V., Balkus K.J., Ferraris J.P., Musselman I.H. (2014). Metal-Organic Polyhedra 18 Mixed-Matrix Membranes for Gas Separation. J. Memb. Sci..

[43] Abeykoon N.C., Bonso J.S., Ferraris J.P. (2015). Supercapacitor Performance of Carbon Nanofiber Electrodes Derived from Immiscible PAN/PMMA Polymer Blends. Rsc Adv..

[44] He D., Xing S., Sun B., Cai H., Suo H., Zhao C. (2016). Design and Construction of Three-Dimensional Flower-like CuO Hierarchical Nanostructures on Copper Foam for High Performance Supercapacitor. Electrochim. Acta.

[45] Indumathi N., Sridevi C., Gowdhaman A., Ramesh R. (2023). Synthesis, Structural Analysis, and Electrochemical Performance of Chitosan Incorporated CuO Nanomaterial for Supercapacitor Applications. Inorg. Chem. Commun..

[46] Liu Y., Cao X., Jiang D., Jia D., Liu J. (2018). Hierarchical CuO Nanorod Arrays in Situ Generated on Three-Dimensional Copper Foam via Cyclic Voltammetry Oxidation for High-Performance Supercapacitors. J. Mater. Chem. A.

[47] Sayyed S.G., Shaikh A.V., Shinde U.P., Hiremath P., Naik N. (2023). Copper Oxide-Based High-Performance Symmetric Flexible Supercapacitor: Potentiodynamic Deposition. J. Mater. Sci. Mater. Electron..

[48] Chen J., Xu J., Zhou S., Zhao N., Wong C.-P. (2015). Facile and Scalable Fabrication of Three-Dimensional Cu(OH)_2_ Nanoporous Nanorods for Solid-State Supercapacitors. J. Mater. Chem. A.

[49] Zhan Y., Bai J., Guo F., Zhou H., Shu R., Yu Y., Qian L. (2021). Facile Synthesis of Biomass-Derived Porous Carbons Incorporated with CuO Nanoparticles as Promising Electrode Materials for High-Performance Supercapacitor Applications. J. Alloys Compd..

[50] Zhu Z., Wei C., Jiang D., Wu X., Lu M. (2022). Design and Synthesis of MOF-Derived CuO/g-C_3_N_4_composites with Octahedral Structures as Advanced Anode Materials for Asymmetric Supercapacitors with High Energy and Power Densities. Mater. Adv..

[51] Li X., Hector A.L., Owen J.R. (2014). Evaluation of Cu_3_N and CuO as Negative Electrode Materials for Sodium Batteries. J. Phys. Chem. C.

[52] Vidyadharan B., Misnon I.I., Ismail J., Yusoff M.M., Jose R. (2015). High Performance Asymmetric Supercapacitors Using Electrospun Copper Oxide Nanowires Anode. J. Alloys Compd..

[53] He X., Mao X., Zhang C., Yang W., Zhou Y., Yang Y., Xu J. (2020). Flexible Binder-Free Hierarchical Copper Sulfide/Carbon Cloth Hybrid Supercapacitor Electrodes and the Application as Negative Electrodes in Asymmetric Supercapacitor. J. Mater. Sci. Mater. Electron..

[54] Marroquin J.B., Rhee K.Y., Park S.J. (2013). Chitosan Nanocomposite Films: Enhanced Electrical Conductivity, Thermal Stability, and Mechanical Properties. Carbohydr. Polym..

[55] Abbas M., Haque S.F.B., Tian Y., Ferraris J.P., Balkus K.J. (2022). Organic–Inorganic Nanohybrids in Supercapacitors. Hybrid Nanomaterials: Biomedical, Environmental and Energy Applications.

[56] Yadav M.S., Sinha A.K., Singh M.N., Kumar A. (2021). Electrochemical Study of Copper Oxide and Activated Charcoal Based Nanocomposite Electrode for Supercapacitor. Mater. Today Proc..

[57] Yadav M.S. (2020). Metal Oxides Nanostructure-Based Electrode Materials for Supercapacitor Application. J. Nanoparticle Res..

[58] Ramesh S., Kathalingam A., Karuppasamy K., Kim H.-S., Kim H.S. (2019). Nanostructured CuO/Co_2_O_4_@ Nitrogen Doped MWCNT Hybrid Composite Electrode for High-Performance Supercapacitors. Compos. Part B Eng..

[59] Yadav M.S. (2020). Fabrication and Characterization of Supercapacitor Electrodes Using Chemically Synthesized CuO Nanostructure and Activated Charcoal (AC) Based Nanocomposite. J. Nanoparticle Res..

[60] Chatterjee S., Ray A., Mandal M., Das S., Bhattacharya S.K. (2020). Synthesis and Characterization of CuO-NiO Nanocomposites for Electrochemical Supercapacitors. J. Mater. Eng. Perform..

[61] Liu Y., Ying Y., Mao Y., Gu L., Wang Y., Peng X. (2013). CuO Nanosheets/RGO Hybrid Lamellar Films with Enhanced Capacitance. Nanoscale.

[62] Tian Y., Abbas M., Haque S.F.B., Tian S., Zhu X., Xiong G., Ferraris J.P., Balkus K.J. (2023). Vanadium Sesquioxide/Nitride Nanostructures in Electrospun Carbon Fibers for High Energy Density Supercapacitors. ACS Appl. Nano Mater..

[63] Mei B.A., Lau J., Lin T., Tolbert S.H., Dunn B.S., Pilon L. (2018). Physical Interpretations of Electrochemical Impedance Spectroscopy of Redox Active Electrodes for Electrical Energy Storage. J. Phys. Chem. C.

[64] Shinde S.K., Mohite S.M., Kadam A.A., Yadav H.M., Ghodake G.S., Rajpure K.Y., Lee D.S., Kim D.Y. (2019). Effect of Deposition Parameters on Spray Pyrolysis Synthesized CuO Nanoparticle Thin Films for Higher Supercapacitor Performance. J. Electroanal. Chem..

[65] Zhang J., Huang R., Dong Z., Lin H., Han S. (2022). An Illumination-Assisted Supercapacitor of Rice-like CuO Nanosheet Coated Flexible Carbon Fiber. Electrochim. Acta.

[66] Bu I.Y.Y., Huang R. (2017). Fabrication of CuO-Decorated Reduced Graphene Oxide Nanosheets for Supercapacitor Applications. Ceram. Int..

[67] Sudhakar Y.N., Hemant H., Nitinkumar S.S., Poornesh P., Selvakumar M. (2017). Green Synthesis and Electrochemical Characterization of RGO–CuO Nanocomposites for Supercapacitor Applications. Ionics.

[68] Wang R., Xu C., Lee J.-M. (2016). High Performance Asymmetric Supercapacitors: New NiOOH Nanosheet/Graphene Hydrogels and Pure Graphene Hydrogels. Nano Energy.

[69] Purushothaman K.K., Saravanakumar B., Babu I.M., Sethuraman B., Muralidharan G. (2014). Nanostructured CuO/Reduced Graphene Oxide Composite for Hybrid Supercapacitors. RSC Adv..

[70] Krishnamoorthy K., Kim S.-J. (2013). Growth, Characterization and Electrochemical Properties of Hierarchical CuO Nanostructures for Supercapacitor Applications. Mater. Res. Bull..

[71] Dubal D.P., Gund G.S., Lokhande C.D., Holze R. (2013). CuO Cauliflowers for Supercapacitor Application: Novel Potentiodynamic Deposition. Mater. Res. Bull..

[72] Moosavifard S.E., El-Kady M.F., Rahmanifar M.S., Kaner R.B., Mousavi M.F. (2015). Designing 3D Highly Ordered Nanoporous CuO Electrodes for High-Performance Asymmetric Supercapacitors. ACS Appl. Mater. Interfaces.

[73] Xu W., Dai S., Liu G., Xi Y., Hu C., Wang X. (2016). CuO Nanoflowers Growing on Carbon Fiber Fabric for Flexible High-Performance Supercapacitors. Electrochim. Acta.

